# Understanding the mode of action of AgroGain^®^, a biostimulant derived from the red seaweed *Kappaphycus alvarezii* in the stimulation of cotyledon expansion and growth of *Cucumis sativa* (cucumber)

**DOI:** 10.3389/fpls.2023.1136563

**Published:** 2023-04-06

**Authors:** Pushp Sheel Shukla, Nagarajan Nivetha, Sri Sailaja Nori, Debayan Bose, Sawan Kumar, Sachin Khandelwal, Alan Critchley, Shrikumar Suryanarayan

**Affiliations:** ^1^ Research and Development Division, Sea6 Energy Private Limited, Centre for Cellular and Molecular Platforms, National Centre for Biological Sciences-Tata Institute of Fundamental Research, Bengaluru, Karnataka, India; ^2^ Verschuren Centre for Sustainability in Energy and the Environment, Sydney, NS, Canada

**Keywords:** *Kappaphycus alvarezii*, seaweed-based biostimulants, plant growth, cucumber, photosynthesis

## Abstract

Seaweed-based biostimulants are sustainable agriculture inputs that are known to have a multitude of beneficial effects on plant growth and productivity. This study demonstrates that Agrogain^®^ (Product code: LBS6), a *Kappaphycus alvarezii*-derived biostimulant induced the expansion of cucumber cotyledons. Seven days treatment of LBS6-supplementation showed a 29.2% increase in area of expanded cotyledons, as compared to the control. LBS6-treated cotyledons also showed higher amylase activity, suggesting starch to sucrose conversion was used efficiently as an energy source during expansion. To understand the mechanisms of LBS6-induced expansion, real time gene expression analysis was carried out. This revealed that LBS6-treated cotyledons differentially modulated the expression of genes involved in cell division, cell number, cell expansion and cell size. LBS6 treatment also differentially regulated the expression of those genes involved in auxin and cytokinin metabolism. Further, foliar application of LBS6 on cucumber plants being grown under hydroponic conditions showed improved plant growth as compared to the control. The total leaf area of LBS6-sprayed plants increased by 19.1%, as compared to control. LBS6-sprayed plants efficiently regulated photosynthetic quenching by reducing loss *via* non-photochemical and non-regulatory quenching. LBS6 applications also modulated changes in the steady-state photosynthetic parameters of the cucumber leaves. It was demonstrated that LBS6 treatment modulated the electron and proton transport related pathways which help plants to efficiently utilize the photosynthetic radiation for optimal growth. These results provide clear evidence that bioactive compounds present in LBS6 improved the growth of cucumber plants by regulating the physiological as well as developmental pathways.

## 1 Introduction

The human population is predominantly dependent on agriculture for food, fiber, biomass, and green energy (Gomiero et al., 2011). Global urbanization and reduced cultivable land put an immense pressure on agricultural practices to provide food for the ever-increasing population. Environmental factors such as high salinity, extreme temperatures and reduced soil fertility are additional challenges to the agricultural system (Gomiero et al., 2011). To feed the ever-increasing population, global agricultural productivity has to be doubled by 2050 (Qin et al., 2011). To improve this situation current practices mainly rely on the use of excessive chemical fertilizers and pesticides, which in turn threaten agricultural sustainability. Additionally, these chemicals are harmful for the entire ecosystem (Shukla and Prithiviraj, 2021). Advancement in molecular biology has contributed to the development of high yielding and stress tolerant plant varieties, but they have not been widely accepted due to regulatory restrictions in several geographies (Agarwal et al., 2013). Therefore, a sustainable biological-based strategy is required to reduce reliance on synthetic fertilizers.

Plant biostimulants are a category of naturally-derived bioactives that had been widely researched for improving plant growth, abiotic stress tolerance and nutrient-use-efficiency (du Jardin, 2015; Jithesh et al., 2019; Shukla et al., 2018b; Shukla et al., 2019; Shukla and Prithiviraj, 2021; Samuels et al., 2022). Seaweeds are found in most of coastal ecosystems and are exposed to highly variable and extreme environmental conditions (Potin et al., 1999; Shukla et al., 2016). To survive such unfavorable conditions, these seaweeds have adapted to produce unique bioactive compounds such as polysaccharides, polyphenols, polyunsaturated fatty acids, and bioactive peptides (Shukla et al., 2019). Seaweed-derived biostimulants are widely being used in sustainable agriculture (Shukla et al., 2019). Many companies, throughout the world, are involved in commercial production of various types of extracts from several seaweed species (Van Oosten et al., 2017; Shukla et al., 2019). Amongst these are the brown seaweeds, e.g., wild harvested *Ascophyllum nodosum*, *Ecklonia maxima*, *Sargassum* spp., and the sustainably cultivated red seaweed *Kappaphycus alvarezii* are predominantly used as raw materials for extract production. Most of the commercially available seaweed-derived biostimulants are from the brown algae, but very few reports have been published from red and green algae (Pereira et al., 2020; Khan et al., 2009; Shukla et al., 2021)

*Kappaphycus alvarezii*, is a cultivated red seaweed largely produced in the Indo-Pacific Ocean and is an industrially important tropical seaweed. It is a carrageenan-bearing (carrageenophyte or euchuematoid) which has been commercially cultivated in Indonesia, India, Philippines, Malaysia, East Africa (Tanazania and Madagascar) and Brazil and areas of the Caribbean (Tan et al., 2012; de Barros-Barreto et al., 2013; Hurtado et al., 2015; Dumilag et al., 2016). *K. alvarezii* extract (carrageenan) is widely used in the food, beverages, nutraceuticals, pharmaceutical, aquaculture industries and most recently in the agricultural industry. Several companies around the world are now exploiting sustainably cultivated *K. alvarezii* for the commercial production of specific types of extracts as plant biostimulants for sustainable agriculture. The phytostimulatory roles of *K. alvarezii*-derived biostimulants is due to the presence of unique oligosaccharides, quaternary ammonium compounds minerals and proteins (Vaghela et al., 2022). Previously published reports confirmed that different types *K. alvarezii*-derived sap collectively improved yield and stress tolerance in wheat, banana, rice, and maize (Layek et al., 2015; Patel et al., 2018; Ravi et al., 2018; Trivedi et al., 2018; Arun et al., 2019; Kumar et al., 2020). The foliar application of *K. alvarezii*-derived biostimulant mitigates the fungicidal stress in rice by regulating antioxidative pathways and the expression of stress-responsive genes (Banakar et al., 2022). *K. alvarezii*-derived biostimulants also improved plant immunity in Arabidopsis and rice by regulating salicylic acid dependent plant defence pathways (Banakar and Kumar, 2020; Roy et al., 2022).

AgroGain^®^ is a commercial formulation prepared from *K. alvarezii* using a patented technology (US 10,358,391 B2) by Sea6 Energy, Private Limited, Bengaluru, India. The foliar applications of AgroGain^®^ improved the plant growth and yield, however the molecular mechanisms of AgroGain^®^-mediated growth promotion is largely unknown. Cucumber (*Cucumis sativus* L.), belongs to family Cucurbitaceae, is an important horticultural crop consumed throughout the world (Shukla et al., 2014). It is used majorly as a common dietary supplement in the form of salad and pickles. In this study, selected molecular mechanisms of AgroGain^®^-mediated expansion of cucumber cotyledon is reported. In addition to that, the effect of foliar spray on AgroGain^®^ on plant growth was evaluated by studying phenotypic, physiological, and photosynthetic parameters.

## 2 Materials and methods

### 2.1 Seaweed extract

### 2.2 Chemical characterisation of AgroGain^®^

In this study, AgroGain^®^ (LBS6), a liquid biostimulant was prepared by blending the liquid and acid hydrolysate of solid fractions of *Kappaphycus alvarezii* extract by following the patented protocols (Nori et al., 2019, US10358391B2). The bioactive ingredients of LBS6 comprises of the sulfated galacto- oligosaccharides of defined molecular weight range (400-10000 Da) (Nori et al., 2019). The chemical composition analysis of LBS6 was performed at Lufa Nord-West (Germany) as an outsourced service and presented as **Supplementary Table S1**.

### 2.3 Cotyledon expansion assay

#### 2.3.1 Plant material and treatments

Seeds of cucumber (*C. sativa*. variety Shiny) were procured from Ascen HyVeg Private Limited (India). The seeds were surface sterilised with 100% ethanol and 2.5% Chlorox^®^ and rinsed with autoclaved distilled water for five times. The sterilized seeds were germinated on tissue paper moistened with 5ml of sterile water. The seeds were incubated in the dark at 25°C. After 5 days, the cotyledons were excised from the etiolated seedlings and placed on filter paper pre-moistened with 5 ml of water (control), or LBS6 solution prepared in water at a concentration of 1 ml/L. This concentration of the LBS6 was selected based on the previously published literature on this product (Ravi et al., 2018; Arun et al., 2019; Nori et al., 2019). The petri dishes containing the cucumber cotyledons were placed in growth chamber (Model No: AR-41L3, Percival, USA) maintained at 24°C with a photoperiod (70% of all spectra using LED panels) of 16/8 h (day/night) and 65% relative humidity (RH). Expansion of the cotyledons was observed over seven days and recorded into a time-lapse video. The time lapse video was recorded by overlapping the pictures clicked at every two minutes by Raspberry pi camera. The expansion of the cotyledon in terms of their total area was recorded at 0, 2, 4 and 7-days after treatment (DAT) by scanning the pictures using EPSON scanner with WinFolia software. The fresh weights of seven-day old cotyledons were recorded. The dry weights of the cotyledons were recorded after drying it in a hot air oven at 70°C for 72 h. The values for the morphological parameters were recorded from three independent experiments. Each experiment consists of six petri dishes with ten cotyledons (60 replicates) and was repeated thrice. For time-lapse video, the expansion of four cotyledons per petri dish were recorded.

#### 2.3.2 Determination of chlorophyll content

Chlorophyll and carotenoid of the seven-DAT cotyledons were determined according to the protocol of Lichtenthaler (1987). Briefly, pigments were extracted from 0.5 gm of cotyledons by instantly grinding with a mortar and pestle in 5 ml of methanol, at 4°C, in 15 ml centrifuge tube. The mixture was centrifuged at 10,000 rpm at 4°C for 10 min, and the pellet was re-extracted with 10 ml of cold methanol until all colour was removed. Two extracts were combined, and the total volume was made up to 15 ml with cold methanol. Absorbance was measured at 470, 652.4, and 665.2 nm using a UV-VIS spectrophotometer (NanoQuant, Tecan, Switzerland). The chlorophyll and carotenoid contents were calculated as described by Lichtenthaler (1987).


$${\text{Chl}}_{\text{a}}=16.72{\text{A}}_{665.2}-9.16{\text{A}}_{652.4}$$

$${\text{Chl}}_{\text{b}}=34.09{\text{A}}_{652.4}-15.28{\text{A}}_{665.2}$$

$${\text{Carotenoids=(1,000A}}_{470}-1.63{\text{Chl}}_{\text{a}}-104.96{\text{Chl}}_{\text{b}})/221$$

#### 2.3.3 Determination of amylase activity

*Qualitative assay:* Effect of the LBS6 on the amylase activity of the cucumber cotyledons was analysed by measuring the area of a clear zone produced by degradation of starch, in solidified starch medium, after treatment with iodine. Briefly, filter-sterilized (0.22 µm SFCA syringe filter) LBS6 (1 ml/L) was added to autoclaved molten starch medium (0.5% soluble potato starch and 0.5% agar) to a final concentration equivalent to 1 mL/L of LBS6. Fifteen-millilitre aliquots of the warm medium were then transferred to 9-cm petri plates and allowed to cool on a level surface. The cucumber cotyledons were placed on the surface of the solidified medium so that the cut ends were in contact with the surface. All procedures were carried out under aspetic conditions in laminar air flow. Three cotyledons were placed in each petri plate and incubated at room temperature (25 ± 2°C) in the dark for 48 h. Following incubation, the cotyledons were removed, and the plates flooded with iodine solution (6 g KI + 0.6 g I_2_ dissolved in 1 L of 0.05 N HCl). The iodine reacted with the starch in the medium to give a blue colour. A clear zone, indicating the hydrolysis of starch, appeared around the cotyledons that had secreted amylase. The area of the cleared zone correlated directly with the amount of amylase secreted by the cotyledons (Ho et al., 1980).

*Quantitative assay:* For the quantification of amylase activity, etiolated cotyledons were kept on petri plates containing autoclaved RO water (Control) and LBS6 (1 ml/L). Samples were collected at 24, 48 and 72 h, after treatment, stored at -80°C. Crude extracts (CE) were prepared according to the method of Juliano and Varner (1969). For the assay of total amylase, 0.1 mL of crude enzyme was added with 0.1 mL of starch solution (150 mg soluble potato starch, 600 mg KH_2_PO_4_ and 294 mg CaCl_2_ in 100 ml distilled water), 0.3 mL of distilled water and incubated at 37°C for 10 min. Another set was prepared as control by adding distilled water instead of starch solution. Immediately after 10 min, the samples were incubated in a boiling water bath for 10 min to stop the reaction. The tubes were cooled, and 0.5 mL of DNS reagent added (1 g NaOH, 1g sodium potassium tartrate and 1 g of dinitro salicylic acid in 100 mL distilled water). The tubes were again incubated at 100°C for 10 min, cooled and absorbance was measured at 540 nm. The glucose content of the sample was measured by comparing it with a glucose standard curve. The conversion of starch, by amylase, to glucose was calculated and expressed as mg of glucose produced/g fresh weight of sample/min.

#### 2.3.4 Gene expression analysis

To understand the role of LBS6-induced expansion of cotyledons, real-time expressions of the genes involved in cell number, division rate, expansion, size, and phytohormonal regulation were carried out using specific primers designed from sequences retrieved from ENSEBL-Plants (**Supplementary Table S2**). For gene expression analysis, the etiolated, excised cotyledons were placed on filter paper moistened with 1ml/L of LBS6 and sterile double distilled water. The cotyledon samples were harvested after 0, 8, 24, 48 and 72 h. The samples were flash frozen in liquid nitrogen and stored at -80°C. Total RNA was extracted using Trizol (Takara Bio, USA), and quantified with NanoDrop One (ThermoScientific, USA). The cDNA was prepared using 1 µg of RNA with iScript cDNA synthesis kit (Bio-Rad, USA). Real-time qPCR analysis was performed in a Quant Studio5 (ThermoScientific, USA). β-tubulin was used as a reference gene. The specificity of PCR amplification was confirmed at the end of the PCR cycles by melt-curve analysis. Three independent experiments were performed with three replicates each, and the relative gene expression was calculated using the 2^-ΔΔCt^ method (Livak and Schmittgen, 2001).

#### 2.3.5 Determination of fructose, and sucrose content from cucumber cotyledons

The fructose and sucrose content was determined colorimetrically following enzymatic hydrolysis using a kit-based assay (Megazyme, Ireland). The cotyledons were carefully excised and placed in round petri-plates with filter paper soaked with autoclaved distilled water (control) or LBS6 at a dosage of 1 ml/L. Cotyledon samples were harvested in liquid nitrogen at 2, 3 and 5 days after treatment. Briefly, fructose and sucrose from cucumber cotyledons was estimated as previously reported (Todaka et al., 2000). The D-glucose content of the total sugar pool was measured following hydrolysis by hexokinase to glucose-6-phosphate (G-6-P) which, subsequently, in the presence of glucose-6-phosphate dehydrogenase (G-6-P DH) is oxidized by nicotinamide-adenine dinucleotide phosphate (NADP^+^) to gluconate-6-phosphate. The reduced nicotinamide-adenine dinucleotide phosphate (NADPH) was measured at 340 nm, which is stoichiometric with the amount of glucose present in the total sample. For the measurement of sucrose, the D-glucose concentration of the total sugars present was determined before and after hydrolysis of the sucrose using β-fructosidase. The sucrose content was calculated based on the difference in absorbance measured at 340 nm. The fructose content was estimated following hydrolysis by hexokinase into fructose-6-phosphate (F-6-P), which is subsequently converted to G-6-P by the enzyme phosphoglucose isomerase (PGI). G-6-P reacts with NADP^+^ to yield gluconate-6-phosphate and NADPH leading to increasing absorbance at 340 nm which was stoichiometric with the amount of fructose present in the sample.

### 2.4 Plant growth assay

The effects of foliar spray of LBS6 on the growth and morphology of cucumber plants were assayed under hydroponic conditions. Seeds of cucumber were surface sterilised and germinated on the filter paper moistened with sterile double distilled water for 5 days. Uniform seedlings were transferred to the thermocol discs floated over 200 ml of ½ Hoagland salt solution (Himedia, India) in plastic Phyta Jars. After seven days of growth, plants were sprayed with 10 ml of 1ml/L of LBS6 with 0.01% Tween 20, and the second spray was made after 7 DAT. The plants were grown for 14 days after the second foliar spray. Entire plant growth assay was carried out in the growth chamber (Model No: AR-41L3, Percival, USA) maintained at 24°C with a photoperiod (70% of all spectra using LED panels) of 16/8 h (day/night) and 65% RH. Fourteen days after second spray of LBS6, increase in the leaf area of the plants were recorded using WinFOLIA software. Plants sprayed with 10 ml of water with 0.01% Tween 20 served as control and were grown under identical conditions as treated plants. Fresh weight of shoots and roots were recorded by weighing on digital analytical balance (Sartorious, Germany). The shoots and roots were separated and dried in a hot air oven at 70°C for 72 h. The data were generated using ten plants for each experimental conditions, and the experiment was repeated thrice (n=30).

#### 2.4.1 Measuring chlorophyll fluorescence

Chlorophyll fluorescence (ChlF) was measured using a handheld MultispeQ V2.0 (PhotosynQ LLC, East Lansing, MI) linked to PhotosynQ platform, using the protocol Photosynthesis Rides 2.0 (www.photosynQ.org). Measurements were taken on the second and third fully expanded leaves of 28 days-old cucumber plants, sprayed twice with 1 ml/L of LBS6, at an interval of seven days. The following ChlF parameters were monitored: SPAD, F_s_ (steady state fluorescence), F_v_’/F_m_’ (efficiency of PSII in the light acclimatized state), Phi2 (quantum yield of PSII electron transport), qL (fraction of PSII open centers), PhiNPQ (the fraction of light dedicated to non-photochemical quenching), NPQ (non-photochemical quenching) and PhiNO (the fraction of energy lost through non-regulated photosynthesis processes).

#### 2.4.2 Determination of electron and proton transport-related processes in light

The effect of the foliar spray on electron transport rate (ETR_PSII_), electrochromic band-shifts (ECSt), proton conductance of chloroplast ATP synthase (gH^+^), estimated proton flux through the thylakoid lumen (vH^+^) which represents rate of ATP synthesis, and photosystem I (PS I) activity were estimated from the second and third leaves of eight plants using MultispeQ V2.0 linked to the PhotosynQ platform following the protocol Photosynthesis Rides 2.0 (www.photosynQ.org). Fluorescence-based electron transport rate (ETR_PSII_) was estimated as described by Ibrahimova et al. (2021). ETR_PSII_ was calculated using the value of quantum yield of PSII obtained by pulse-amplitude modulation method at photosynthetically active radiation (PAR) of 500 μmol photons m^-2.^s^-1^ as follows:


$${\text{ETR}}_{\text{PSII}}=0.84*0.5*\text{Phi}2*\text{PAR}$$

### 2.5 Statistical analyses

Data were analysed using one way ANOVA, to calculate the least significant difference, at a probability of 0.05% using WASP 2.0 statistical package from ICARGOA (https://ccari.icar.gov.in/wasp2.0/index.php). When significant effects of treatments were found, multiple means comparison was carried out using Duncan’s multiple range test with 95% confidence interval. The significantly different mean values were represented by different letters.

## 3 Results

### 3.1 Cotyledon expansion assay

#### 3.1.1 LBS6 treated cotyledons induced higher expansion of excised cotyledons

**Figure 1:**
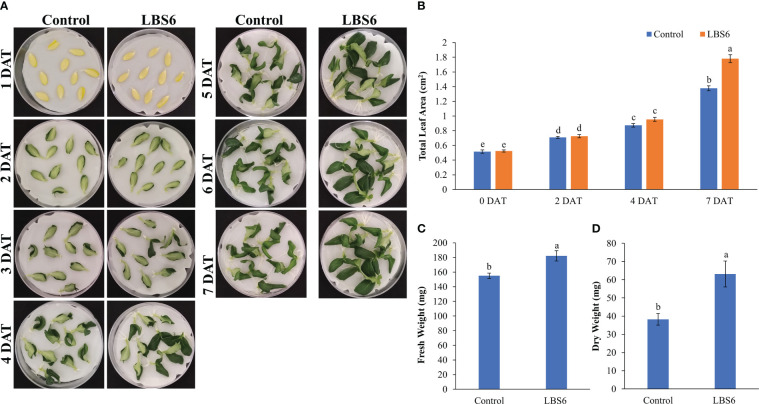
Cotyledon expansion assay **(A)** Effect of LBS6 on expansion of cotyledons. Cotyledons treated with sterile distilled water served as control. **(B)** Total area of seven-day old cotyledons treated with water (Control) and LBS6. Fresh **(C)** and dry **(D)** weight of the seven-day old cotyledons treated with water (Control) and LBS6. The values were presented as mean ± SE of three independent replicates, and significantly different mean values were represented by different letters.

Excised cotyledons were placed on Whatman filter paper moistened with control and 1ml/L of LBS6. The time-lapse video suggests that LBS6 induces the early expansion of the cucumber cotyledons (**Video 1**). After four days of treatment, both the degree of green (pigments) and leaf expansion were greater in the LBS6-treated cotyledons as compared to control (**Figure 1A**) and achieved maximum expansion at day 7. The leaf area of those cotyledons treated with LBS6 was significantly higher (29%) than the control (**Figure 1B**). LBS6-treated cotyledons showed 17.5 and 65.1% higher fresh and dry biomass, respectively, as compared to control (**Figures 1C, D**).

#### 3.1.2 Effect of LBS6 treatment on the pigment content of excised cucumber cotyledons

**Figure 2:**
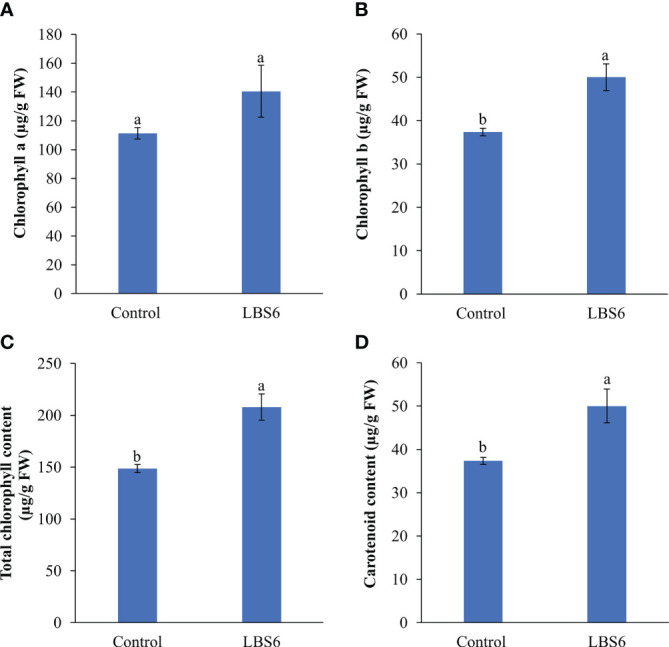
LBS6 improved the pigment content of treated cotyledons. **(A)** chlorophyll a, **(B)** chlorophyll b, **(C)** total chlorophyll, and **(D)** carotenoid content of seven-day old cotyledons treated with water (Control) and LBS6. The values were presented as mean ± SE of three independent replicates, and significantly different mean values were represented by different letters.

LBS6-treated cotyledon has an increased chlorophyll a content, as compared to control, however the difference was not statistically significant (at p=0.05; **Figure 2A**). The chlorophyll b and total chlorophyll contents were significantly higher in LBS6-treated cotyledons, as compared to the water controls (**Figures 2B, C**). The content of carotenoids was also found to be 31.0% higher in the treated cotyledons, as compared to control (**Figure 2D**).

#### 3.1.3 Effect of LBS6-treatment on amylase activity, sucrose and fructose content of cucumber cotyledon

**Figure 3:**
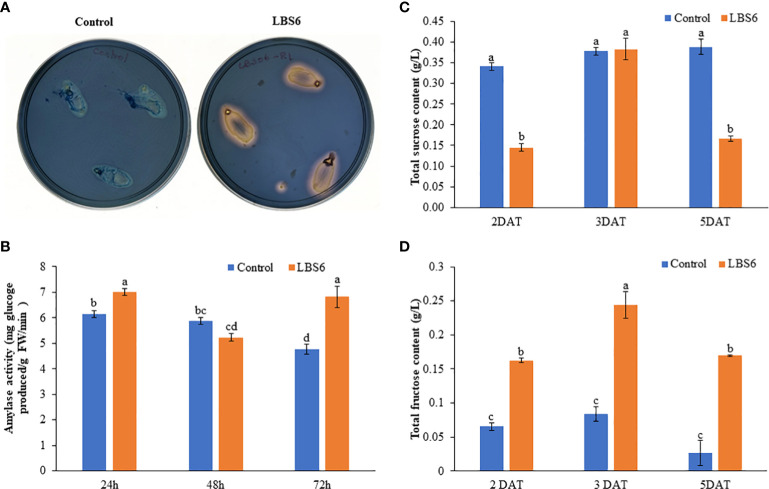
LBS6 modulated amylase activity and sucrose content. **(A, B)** Qualitative and quantitative amylase content in the cotyledon of cucumber. **(C)** Total sucrose, and **(D)** fructose content in the water (Control) and LBS6-treated cotyledons. The values were presented as mean ± SE of three independent replicates, and significantly different mean values were represented by different letters.

The cotyledons placed on 0.8% agar treated with LBS6 secreted more amylase which converted starch to glucose. This was qualitatively estimated by staining the agar plates grown with cotyledons by Lugol’s staining (**Figure 3A**). LBS6-amended agar plates showed more solubilization of starch by amylase around the cotyledon, as compared to control. The amylase activity in the treated cotyledons was measured by estimating the amount of glucose produced by the amylase activity, per unit time. At 24 h, the amylase activity was found significantly higher in LBS6-treated cotyledons, while after 48 h of treatment, the amylase activity was slightly reduced in LBS6-treated cotyledons, as compared to control. Treated cotyledons showed 42.6% higher amylase activity than the control after 72 h of incubation (**Figure 3B**). The sucrose content in treated cotyledon was increased by 164% during the growth from 2 day to 3 day, whilst, in control over same duration, the sucrose content was increased by 10.78% (**Figure 3C**). At 5 DAT, there is reduction in sucrose content in LBS6 treated cotyledons. Fructose content was found to be significant higher in treated cotyledons at all time points (**Figure 3D**). These results showed that the bioactive compounds present in LBS6 induced a better conversion of starch to simple sugars, i.e., as an available energy source for the expanding cotyledons (**Figure 3**).

#### 3.1.4 LBS6 regulated the expression of genes involved in cell division, expansion, total number, and size

**Figure 4:**
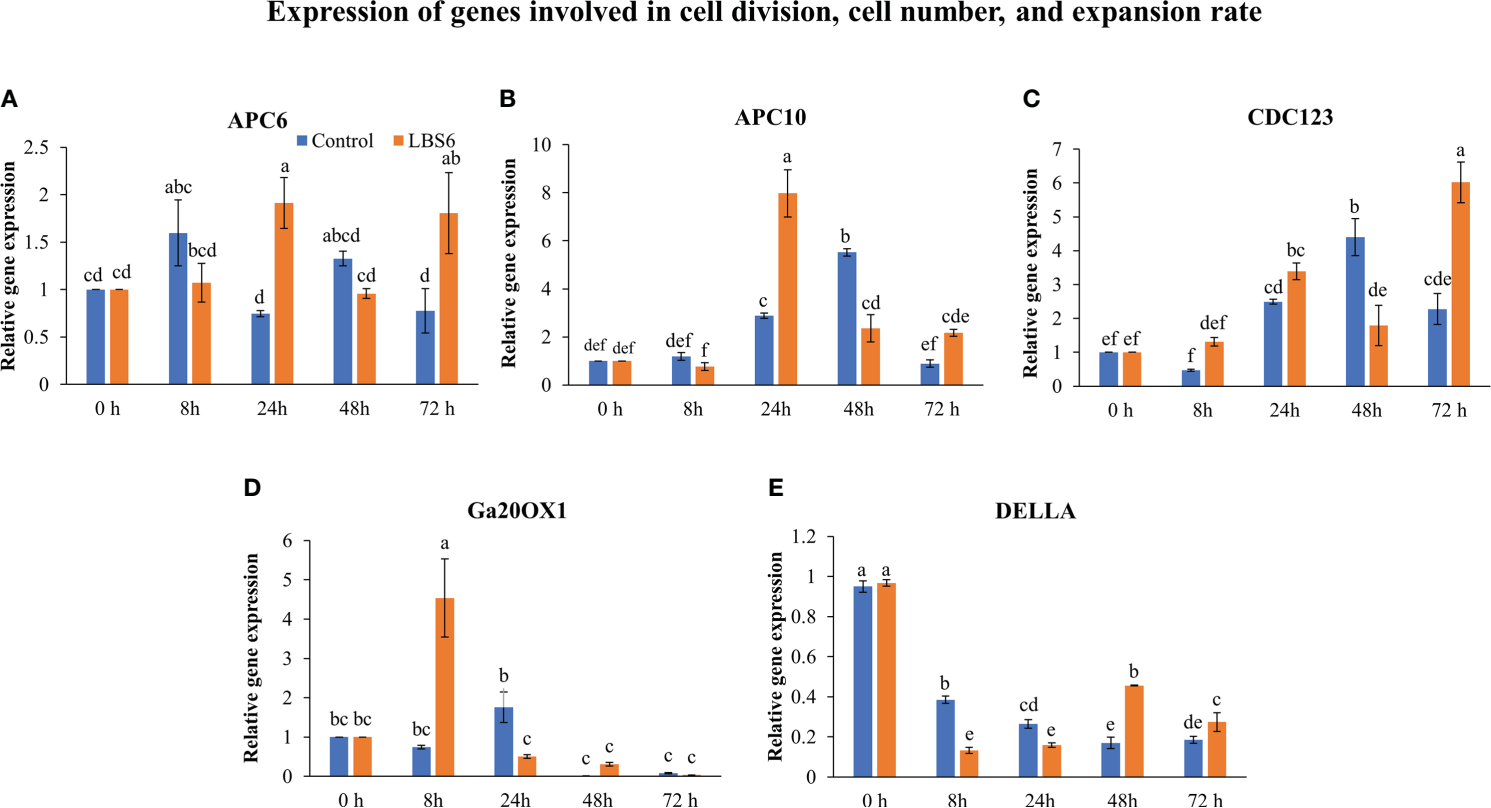
LBS6 regulated the expression of **(A)** *APC6*, **(B)** *APC10*, **(C)** *CDC123*, **(D)** *Ga20OX1*, and **(E)** *DELLA* involved in cell division, and cell number. The value in the graph represents the relative gene expression. The values were presented as mean ± SE of three independent replicates, and significantly different mean values were represented by different letters.

The application of LBS6 induced the expansion of the cotyledon. To elucidate the molecular mechanism of induction of cotyledon expansion by LBS6, the expressions of different genes involved were studied. Anaphase-promoting complex (APCs) controls the cell cycle progression resulting in formation of enlarged leaves (Vercruysse et al., 2020). In this study, the cotyledons treated with LBS6 showed that *APC6* was differentially expressed at different time points. Initially, after 8 h, *APC6* expression was slightly less in LBS6-treated seedlings than the control, but at 24 h it was significantly higher in treated seedlings (**Figure 4A**). Twenty-four hours after treatment, expression of *APC10* was 2.8 times higher in those LBS6-treated cotyledons than the control (**Figure 4B**). No significant difference in the expression of *CDC123* (cell division cycle protein 123 homolog) was observed 24 h after treatment, whilst at 48 h there was a reduction in transcripts of *CDC123* in LBS6-treated cotyledons (**Figure 4C**). Seventy-two hours after treatment, expression of *CDC123* was 2.6 times higher in the treated cotyledons (**Figure 4C**). Gibberellic acid plays a crucial role in cell proliferation and division (Achard et al., 2009). The expression of gibberellin 20-oxidase 1 (GA20OX1), an enzyme involved in gibberellic acid biosynthesis, was significantly induced by LBS6 8 h after treatment (**Figure 4D**). DELLA proteins negatively regulate plant growth by inhibiting division and expansion of cells (Gonzalez et al., 2010). Till 24 h, LBS6 treatment down-regulated *DELLA* transcripts in treated cotyledons, as compared to the control (**Figure 4E**). At 48 and 72 h time-point the expression of DELLA was induced in LBS6 treated cotyledon (**Figure 4E**).

**Figure 5:**
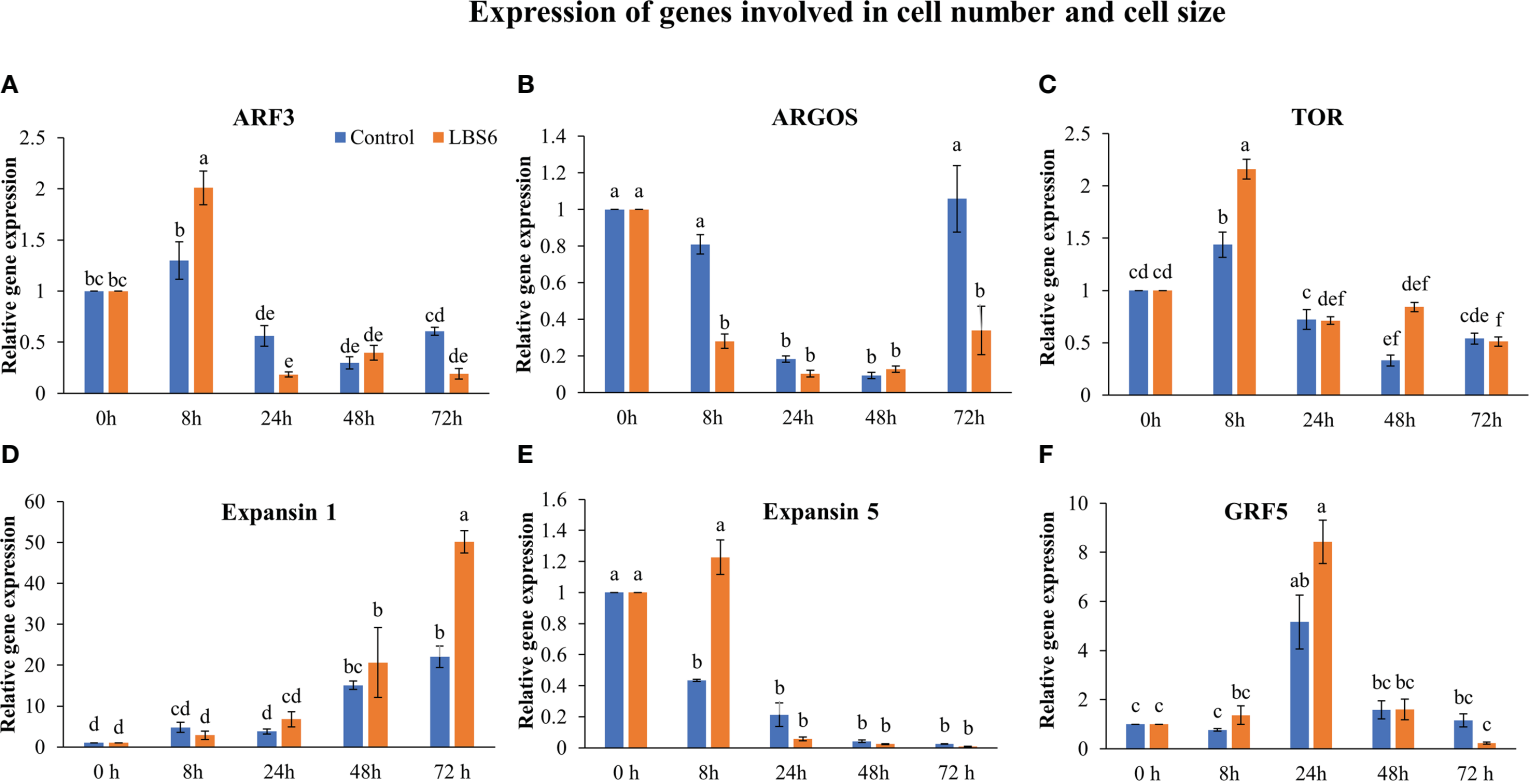
LBS6 regulated the expression of **(A)** *ARF3*, **(B)** *ARGOS*, **(C)** *TOR*, **(D)** *Expansin 1*, **(E)** *Expansin 5*, and **(F)** *GRF5* involved in cell size, and cell expansion. The values in the graph represents the relative gene expression. The values were presented as mean ± SE of three independent replicates, and significantly different mean values were represented by different letters.

Auxin response factors (ARFs) negatively regulates cell division and expansion and it was differentially regulated by LBS6. An interesting expression pattern was observed for *ARF3*. At 8 h, its expression increased in the treated cotyledons, as compared to control. In contrast, at 24 and 72 h, significant reduction was observed in the expression of *ARF3*, in both, control, as well as LBS6-treated cotyledons. Notably, reduction in expression of this gene clearly demonstrated that LBS6 treatment induced cell division and expansion by down-regulating the expression of *ARF3* (**Figure 5A**). Auxin regulated gene involved in organ size (*ARGOS*) was significantly down-regulated in LBS6-treated cotyledons at 8 h and 72 h, as compared to the control. However, at 24 h and 48 h, no changes in the expression of *ARGOS* was observed in either LBS6-treated or control cotyledons (**Figure 5B**). Target of rapamycin (TOR), a serine/threonine protein kinase, is a key regulator of cell growth, proliferation and expansion (Ahmad et al., 2019). At 8 h, LBS6-treated cotyledons showed a significantly higher abundance of transcripts of TOR, as compared to the control (**Figure 5C**). The expression of *Expansin1* (*Exp1*), involved in cell expansion by activating a non-enzymatic, pH dependent, loosening and softening of cell walls (Marowa et al., 2016), was found to be significantly higher in treated cotyledons, after 72 h, as compared to control (**Figure 5D**). In control cotyledons, the abundance of *Exp5* decreased spontaneously till 72 h, while in LBS6-treated cotyledons, this gene was induced at 8 h, and then its expression was reduced spontaneously until 72 h (**Figure 5E**). Growth regulating factor 5 (*GRF5*), promotes the duration of cell proliferation during leaf development and was found to be significantly induced at 24 h in treated cotyledons (**Figure 5F**). Taken together these results collectively suggest that the application of LBS6 significantly improved cell proliferation and expansion in treated cotyledons, which in turn promoted leaf growth.

#### 3.1.5 LBS6 application modulated the expression of genes involved in auxin metabolism

**Figure 6:**
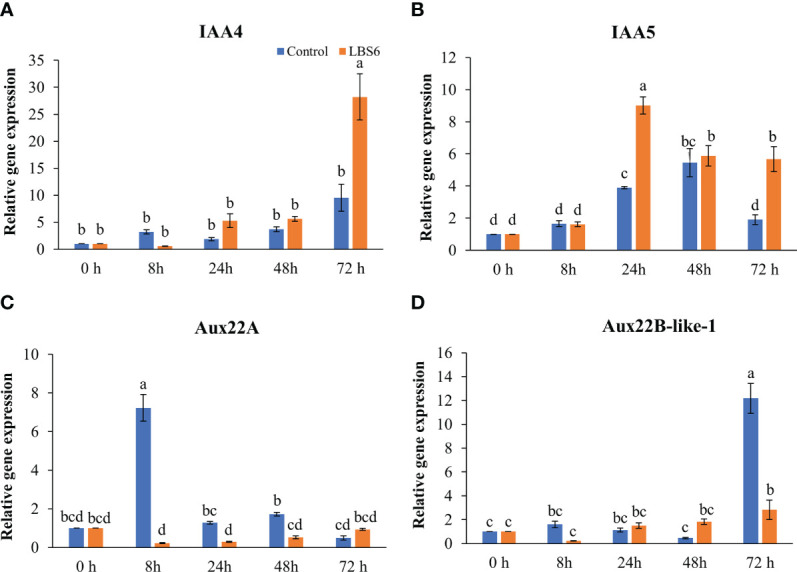
LBS6 modulate the expression of **(A)** *IAA4*, **(B)** *IAA5*, **(C)** *Aux22A*, and **(D)** *Aux22B-like-1* involved in auxin biosynthesis. The values in the graph represents the relative gene expression. The values were presented as mean ± SE of three independent replicates, and significantly different mean values were represented by different letters.

Auxins plays major roles in cell expansion and differentiation by regulating gene expression (Tian et al., 2002). At 8, 24, and 48 h of treatment, no change in the expression of indole acetic acid 4 (IAA4) was observed (**Figure 6A**). At 72 h, the expression of IAA4 was 3-fold higher in LBS6-treated cotyledons, as compared to control (**Figure 6A**). The abundance of the transcript for *IAA5* was 2.3-fold higher in treated cotyledons than respective control after 24 h of treatment. This trend then reduced in both LBS6-tretaed and control, but reduction was greatest in the control samples (**Figure 6B**). *Aux22A (Auxin induced protein 22A)* is a negative regulator of auxin-mediated gene responses and was significantly down-regulated in LBS6 treated cotyledons, as compared to respective controls at 8 and 24 h (**Figure 6C**). There were no significant changes in expression of *Aux22B*-like-1 up to 48 h of treatment. At 72 h, a distinct increase in the abundance of the transcript of *Aux22B*-like-1 was observed in the control, whilst the expression of this gene was 6-fold less in the LBS6-treated cotyledons, as compared to control (**Figure 6D**). These results indicated that LBS6 treatment modulated expression of genes involved in auxin metabolism and thereby regulated expansion of cucumber cotyledons.

#### 3.1.6 LBS6 modulated expression of genes involved in cytokinin metabolism

**Figure 7:**
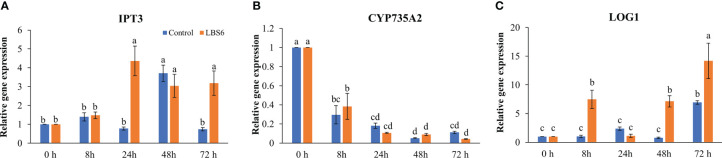
LBS6 modulated the expression of **(A)** *IPT3*, **(B)** *CYP735A2*, and **(C)** *LOG1* involved in cytokinin biosynthesis pathways. The values in the graph represents the relative gene expression. The values were presented as mean ± SE of three independent replicates, and significantly different mean values were represented by different letters.

Cytokinin plays an important role in cell differentiation. In our study, the effect of LBS6 on cytokinin metabolism during cotyledon growth was analyzed. The isopentenyl transferase 3 gene (*IPT3*) is important in cytokinin biosynthesis, it was found to be significantly induced in those cotyledons treated with LBS6 after 24 h, while in control cotyledons maximum induction was observed after 48 h. This showed that LBS6 treatment regulated the early induction of *IPT3* in treated cotyledons (**Figure 7A**). The abundance of the *CYP735A2* transcript was found to be lower in both the control and LBS6-treated cotyledons at all time-points (**Figure 7B**). No significant changes were observed in the expression of *CYP735A2* in either control or treated cotyledons, at all-time points (**Figure 7B**). It was observed that the Lonely Guy 1 (*LOG1*) gene, known to activate adenylate-type pre-cytokinins, was significantly induced in LBS6-treated cotyledons, in a time-dependent manner. At 8 h, treated cotyledons showed a 7.3-fold higher expression of *LOG1*, however expression of this gene was reduced to 2.1-fold in LBS6-treated cotyledons, as compared to control (**Figure 7C**). At 48 h, expression of *LOG1* increased by 9-fold in the treated cotyledons, as compared to their respective controls (**Figure 7C**). The highest expression of *LOG1* was observed after 72 h, in both control and treated cotyledons, but LBS6-treated plants were 2-fold higher than their control (**Figure 7C**).

**Figure 8:**
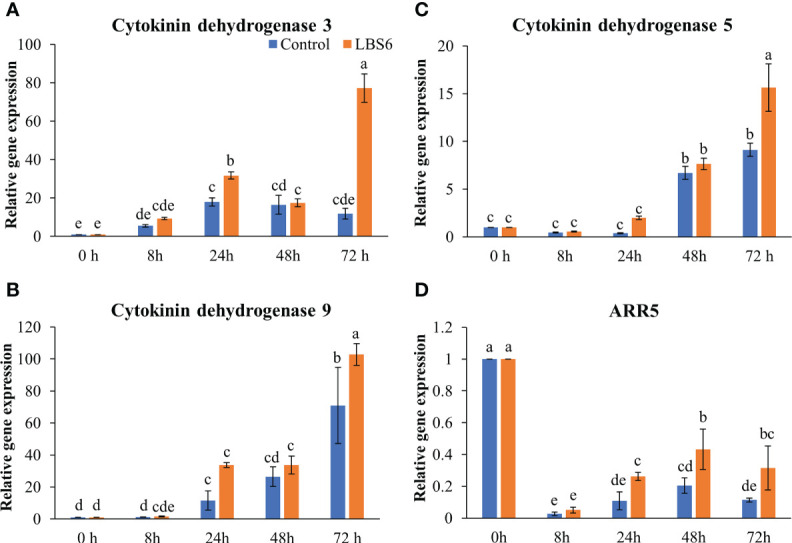
LBS6 modulated the expression of **(A)** *Cytokinin dehydrogenase 3*, **(B)** *Cytokinin dehydrogenase 9*, **(C)** *Cytokinin dehydrogenase 5*, and **(D)** *ARR5* involved in cytokinin degradation pathways. The values in the graph represents the relative gene expression. The values were presented as mean ± SE of three independent replicates, and significantly different mean values were represented by different letters.

Cytokinin dehydrogenases plays important degrading roles in cytokinin metabolism (Schmülling et al., 2003; Gu et al., 2010). Cytokinin dehydrogenase 3 and Cytokinin dehydrogenase 9 were significantly induced at 24 and 72 h in treated cotyledons, as compared to the controls (**Figures 8A, B**). The transcript abundance of *Cytokinin dehydrogenase 5* was found to be 1.7 times higher in treated cotyledons (**Figure 8C**). The A-type Arabidopsis Response regulator 5 (*ARR5*), which acts as the primary cytokinin negative feedback regulator was down-regulated in all treatments and controls, at all the time points. Indubitably reduced expression of this gene was greatest in the control cotyledons (**Figure 8D**). These results further demonstrated that LBS6 treatments modulated expression of genes involved in cytokinin metabolism in the cucumber.

### 3.2 Plant growth assay

#### 3.2.1 LBS6 improved plant growth by increasing leaf area and root volume

**Figure 9:**
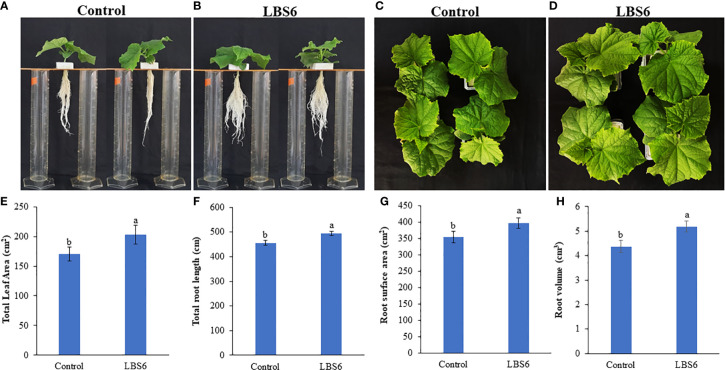
*Kappaphycus alvarezii* based extract (LBS6) improved the growth of cucumber plants. Twenty eight day old plants sprayed with **(A, C)** water (Control), and **(B, D)** LBS6. Here the effects of LBS6 on **(E)** total leaf area, **(F)** total root length, **(G)** root surface area, and **(H)** root volume of cucumber plants are shown. The values were presented as mean ± SE and means represented by the same letters were not significantly different at p ≤ 0.01. Each experiment was carried out in triplicate, and each experimental unit had ten plants (n = 30).

**Figure 10:**
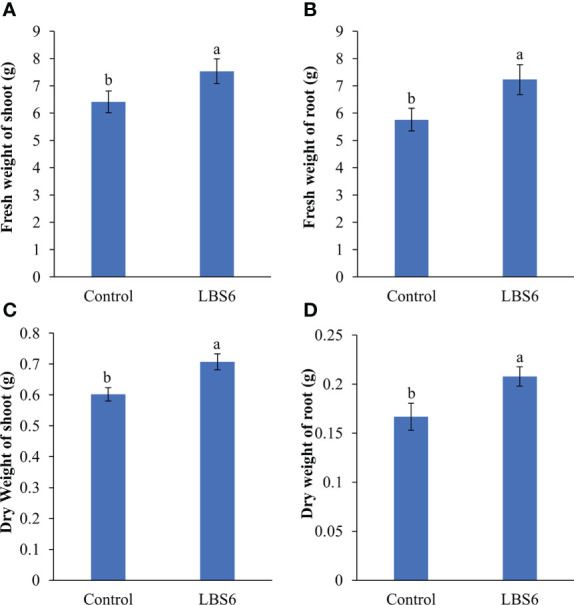
Effect of LBS6 on fresh and dry weight of **(A, C)** shoot, and **(B, D)** root, respectively. The values were presented as mean ± SE and means represented by the same letters were not significantly different at p ≤ 0.01. Each experiment was carried out in triplicate, and each experimental unit had ten plants (n = 30).

Foliar spray of 1ml/L of LBS6 enhanced the morphological phenotype of cucumber plants (**Figures 9A–D**). The treated plants had 20% higher leaf area, as compared to control (**Figure 9E**). The plants sprayed with LBS6 showed enhanced root architecture, compared to control, i.e., total root length was 8.5% longer in 28 days grown plant under hydroponic conditions (**Figure 9F**). The surface area and root volume were increased by 6.5% and 18.6%, respectively in the LBS6-treated plants versus control (**Figures 9G, H**). The fresh biomass of the shoots and roots were statistically higher in the LBS6 sprayed plants (**Figures 10A, B**). The dry biomass of shoot and root was found statistically higher in the sprayed plants, as compared to control (**Figures 10C, D**).

#### 3.2.2 Photosynthetic responses to LBS6 treatments

Soil Plant Analyser Development (SPAD) values can be directly correlated to the chlorophyll content of leaves (Kandel, 2020). SPAD values were found to be significantly higher in LBS6-sprayed plants, as compared to control (**Table 1**). LBS6-treated plants showed higher active and open PSI centres, but the increment in those values was not statistically significant (**Table 1**). PSI were more oxidised in the LBS6-treated (1.48) versus control plants (0.92). The control plants had a greater reduction of PSI centres (-0.48) than the LBS6-sprayed plants (-1.85) (**Table 1**). The data related to chlorophyll fluorescence at steady state (Fs), the efficiency of PSII in the light acclimatized state (F_v_’/F_m_’), fraction of PSII open center (qL) and quantum yield of PSII electron transport (Phi2) did not show significant changes in LBS6 treated and control plants. However, the values of NPQ (non-photochemical quenching) and PhiNPQ (the fraction of light dedicated to non-photochemical quenching) that represents the loss of the incident photosynthetic light in form of thermal dissipation is significantly less in LBS6-sprayed plants, as compared to control (**Table 1**). No significant change was observed in the fraction of the energy lost through non-regulated photosynthesis processes in the sprayed plants (**Table 1**).

**Table 1:** Effect of LBS6 on the chlorophyll fluorescence parameters from leaves of the cucumber plants.

S.No.	Parameters	Control	LBS06
1.	SPAD	54.13 ± 0.72^b^	57.12 ± 0.63^a^
2.	PS1 active centre	0.81 ± 0.05^a^	01.02 ± 0.02^a^
3.	PS1 Open centre	0.57 ± 0.08^a^	01.44 ± 0.34^a^
4.	PS1 oxidised centre	0.92 ± 0.15^a^	01.48 ± 0.15^b^
5.	PS1 reduced centre	-0.48 ± 0.13^a^	-1.85 ± 0.60^b^
6.	F_s_ (steady state fluorescence)	1291.88 ± 15.11 ^a^	1257.58 ± 08.89 ^a^
7.	${\text{F}}_{v}^{'}/{\text{F}}_{m}^{'}$ (Efficiency of PSII in light acclimatized state)	0.69 ± 0.008 ^a^	0.72 ± 0.01 ^a^
8.	Phi2 (Quantum yield of PSII)	0.61 ± 0.01 ^a^	00.62 ± 0.003 ^a^
9.	qL (Fraction of PSII open center)	0.70 ± 0.01 ^a^	0.68 ± 0.01 ^a^
10.	NPQ_t_ (Non-photochemical quenching)	1.15 ± 0.024 ^a^	1.05 ± 0.021^b^
11.	PhiNPQ (Fraction of light involved in non-photochemical quenching)	0.207 ± 0.004 ^a^	0.192 ± 0.003 ^b^
12.	PhiNO (Fraction of energy lost through non-regulated photosynthesis processes)	0.179 ± 0.001 ^a^	0.173 ± 0.01^a^

The values were presented as mean ± SE and means represented by the same letters in superscript were not significantly different at p ≤ 0.01.

#### 3.2.3 Effect of LBS6 application on electron and proton transport-related processes in light-adapted state in leaves of cucumber plants

**Figure 11:**
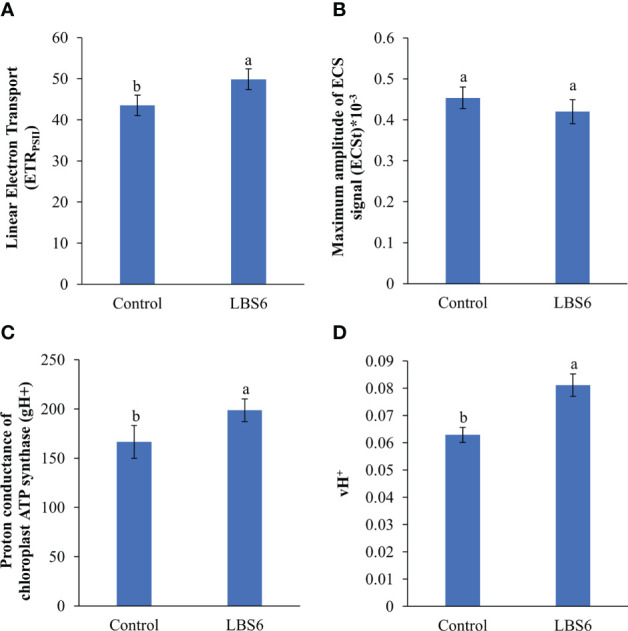
Effect of LBS6 on **(A)** linear electron transport, **(B)** maximum amplitude of ECS signal (ECS_t_), **(C)** proton conductance of chloroplast ATP synthase (gH^+^), and **(D)** estimated proton flux through thylakoid lumen (vH^+^). The values were presented as mean ± SE and means represented by the same letters were not significantly different at p ≤ 0.01. Each experiment was carried out in triplicate, and each experimental unit had ten plants (n = 30).

The rate of linear electron transport was estimated from the quantum yield of PSII in the light adapted state (ETR_PSII_), and was found to be higher in those LBS6-sprayed plants, as compared to the control (**Figure 11A**). The effect of treatment on the proton transport, at the thylakoid membrane was measured using electrochemical band shift analysis at 520 nm, applying dark-interval relaxation kinetic analysis. The maximum amplitude of the signal (ECS_t_) was reduced in the LBS6-sprayed plants, but the change was not statistically significant (**Figure 11B**). The proton conductance of chloroplast ATP synthase (gH^+^) was found to be 19.4% higher in the LBS6 sprayed leaves, as compared to the control (**Figure 11C**). Similarly, the estimated proton flux through the thylakoid lumen (vH^+^), was used to calculate the relative flux through H^+^-ATP-ase which was found to be significantly higher in LBS6-treated plants (**Figure 11D**).

## 4 Discussion

Climatic challenges and nutrient deprivation of the soils effects the plant growth and yield. Current agricultural practices mainly rely on the usage of chemical fertiliser for improving agricultural productivity. The over usage of these fertilisers poses threats to the entire ecosystem and human health. Seaweed-based, agricultural inputs (phyco-biostimulants, as opposed to other biostimulants) - are gaining interest as a sustainable strategy to improve plant growth and stress tolerance (El Boukhari et al., 2020; Shukla et al., 2021; Deolu-Ajayi et al., 2022). Seaweed-based biostimulants are known to improve plant growth, nutrient use efficiency and abiotic and biotic stress tolerance in several crops (Nair et al., 2012; Shukla et al., 2018a; Di Stasio et al., 2018; Jithesh et al., 2019; Sandepogu et al., 2019; Kumar et al., 2020; Omidbakhshfard et al., 2020; Shukla and Prithiviraj, 2021; Trivedi et al., 2021). This study presents the molecular and physiological effects of foliar spray of unique red seaweed derived biostimulant (LBS6) on plant growth.

### 4.1 Cotyledon expansion assay

A reliable bioassay plays an important role in development of new formulations. Anwar and Shahida (2009) have previously shown that the cytokinin-producing bacteria exhibit positive roles in expansion of cucumber cotyledons. Similarly, Kataria and Guruprasad (2005) had used cucumber cotyledon bioassay to show the effect UV-B radiation cell expansion by modulating cytokinin biosynthesis. We have used this bioassay to demonstrate the effect of a particular red seaweed, *Kappaphycus alvarezii*-derived, (phyco)biostimulant on plant cell growth and expansion. The time-lapse video of the cucumber cotyledon elegantly showed that the LBS6 extract induced cotyledon expansion, which was visually presented (**Video 1**). The total surface area of the expanded cotyledons was significantly larger in the LBS6-treated cotyledons (**Figures 1A, B**). The fresh and dry biomass of LBS6-treated cotyledons were significantly higher than the control (**Figures 1C, D**). The cotyledon grown in LBS6 produced higher chlorophyll and carotenoid contents (**Figure 2**). These results clearly demonstrated that the bioactive compounds present in LBS6 induced cell division, proliferation, and expansion in treated cucumber cotyledons.

Anaphase promoting complex (APC), an E3 ligase, regulates the cell cycle progression (Vercruysse et al., 2020). Eloy et al. (2011) showed that the over-expression of *APC10* led to the enhanced division of epidermal cells and enlarged leaves. In another study, down-regulation of *APC6* and *APC10* showed reduced cell surface area (Marrocco et al., 2009). LBS6-treated cotyledons showed maximum up-regulation of *APC10* after 24 h of treatment, whilst *APC6* was significantly up-regulated at 24 and 72 h. These results showed that cells divide more quickly in LBS6-treated cotyledons than their respective controls by inducing expression of *APC6* and *APC10*, resulting in expansion of the cotyledon. Similarly, *CDC123* - encoding another subunit of the APC complex was induced by LBS6 and increased plant size by regulating the rate of cell division. Gibberellic acid (GA) promotes plant growth by controlling cell proliferation and cell expansion by negatively regulating the expression of *DELLA* protein (Achard et al., 2004; Vercruysse et al., 2020). Over-expression of *GA20OX1*, a key enzyme in GA biosynthesis, induces leaves enlargement by reducing the expression of *DELLA* proteins (Achard et al., 2004; Gonzalez et al., 2010; Vercruysse et al., 2020). LBS6 improved cell proliferation in treated cotyledons by inducing the expression of *Ga20OX1*, leading to higher active GA which mediates the reduction of DELLA protein (**Figures 4D, E**). Therefore, these results provide clear evidence that LBS6 regulated the expression of the genes involved in cell division and cell proliferation.

Sugars are the main source of carbon and energy source and play a prominent role in regulating cell proliferation and expansion (Halford et al., 2011; Eveland and Jackson, 2012). In general, cell expands by accumulating sugar and osmolytes which creates a lower osmotic potential to provide a gradient for water influx, generating a turgor pressure, leading to cell expansion (Wang and Ruan, 2013). Starch is the main carbohydrate reserve in plant cotyledons which is converted by amylase to simple sugars, as source of carbon for leaf expansion (Pfeiffer and Kutschera, 1996; Kutschera and Heiderich, 2002). LBS6-treated plants showed a higher amylase activity suggesting higher conversion of starch to simple sugars. Sucrose is converted to the fructose by invertases (Halford et al., 2011). The percentage increase in sucrose content of LBS6-treated cotyledons during the growth was higher than those of the control during early time points, whilst at later time point there was reduction of sucrose content in LBS6 treated plants. This trend can be explained by higher fructose content in LBS6 treated cotyledons as compared to control, suggesting that at later time point of the growth of cotyledon, more sucrose was converted to fructose. Higher fructose in treated cotyledons were more efficiently supplied for glycolysis, respiration and secondary product formation (Halford et al., 2011). These data provide new insights that LBS6 cotyledon efficiently converted starch to simple sugars by regulating amylase activity which, in turn, provided more energy to the cotyledon expansion. Sugars not only serves as energy source, but also acts as a signalling molecule and regulates the expression of the genes involved in phytohormone biosynthesis, cell division and expansion (Wang and Ruan, 2013). The higher levels of sucrose in the LBS6-treated cotyledons would interact with auxin in order to regulate cell division and leaf expansion. Trehalose-6-phosphate (T6P), a downstream signalling component of carbohydrate metabolism, negatively regulates the expression of sucrose non-fermenting1 (*Snf1*)-related protein kinase (*SnRK1*) and plays an important role in cell proliferation (Wang and Ruan, 2013). Increased sucrose content in the LBS6-treated cotyledons led to the degradation of *Aux22A* and *Aux22B-like-1* protein, which interacts with *ARFs*, so as to suppress auxin-regulated gene expression. Increased auxin content in LBS6-treated cotyledons was evidenced by the higher expression of early auxin inducible IAA4/5 (Abel et al., 1995). Additionally, higher reducing sugars in the LBS6-treated cotyledons provided more cellular energy and nutrients to the cotyledons by inducing the expression of target of rapamycin (TOR), which is known to promote cell proliferation and growth by integrating nutrient and energy signaling (Wang and Ruan, 2013; Xiong and Sheen, 2014).

Auxin induces cell expansion by negatively regulating expression of transcriptional regulators, *ARFs*, which are also known to be regulated by hexose sugars (Wang and Ruan, 2013; Majda and Robert, 2018). Initially, at 8 h LBS6 treatments showed a higher expression of ARF3 than their controls and then, at later time points, a higher reduction in expression of this gene was observed in LBS6-treated cotyledons. This clearly indicated that LBS6 down-regulated ARF*3* leading to higher cell proliferation. Similarly, auxin regulated gene involved in organ size (*ARGOS*) was also found to be down-regulated by the LBS6-treatment, though it is positively regulating the cell expansion and control organ size (Zhao et al., 2017). Auxins are also known to positively regulate expansin proteins involved in non-enzymatic relaxation of the cell wall in a pH dependent manner (McQueen-Mason et al., 1992; Marowa et al., 2016). Cell wall loosening, mediated by expansins led to the expansion of cell wall and growth (Marowa et al., 2016). LBS6 treatments induced the expression of *Expansin 1* and *Expasin 5* which regulated expansion in the cotyledons by changing cell wall plasticity. Thus, LBS6 applications induced cell proliferation and expansion by maneuvering the expression of genes involved in cell expansion, sugar and auxin signaling.

**Figure 12:**
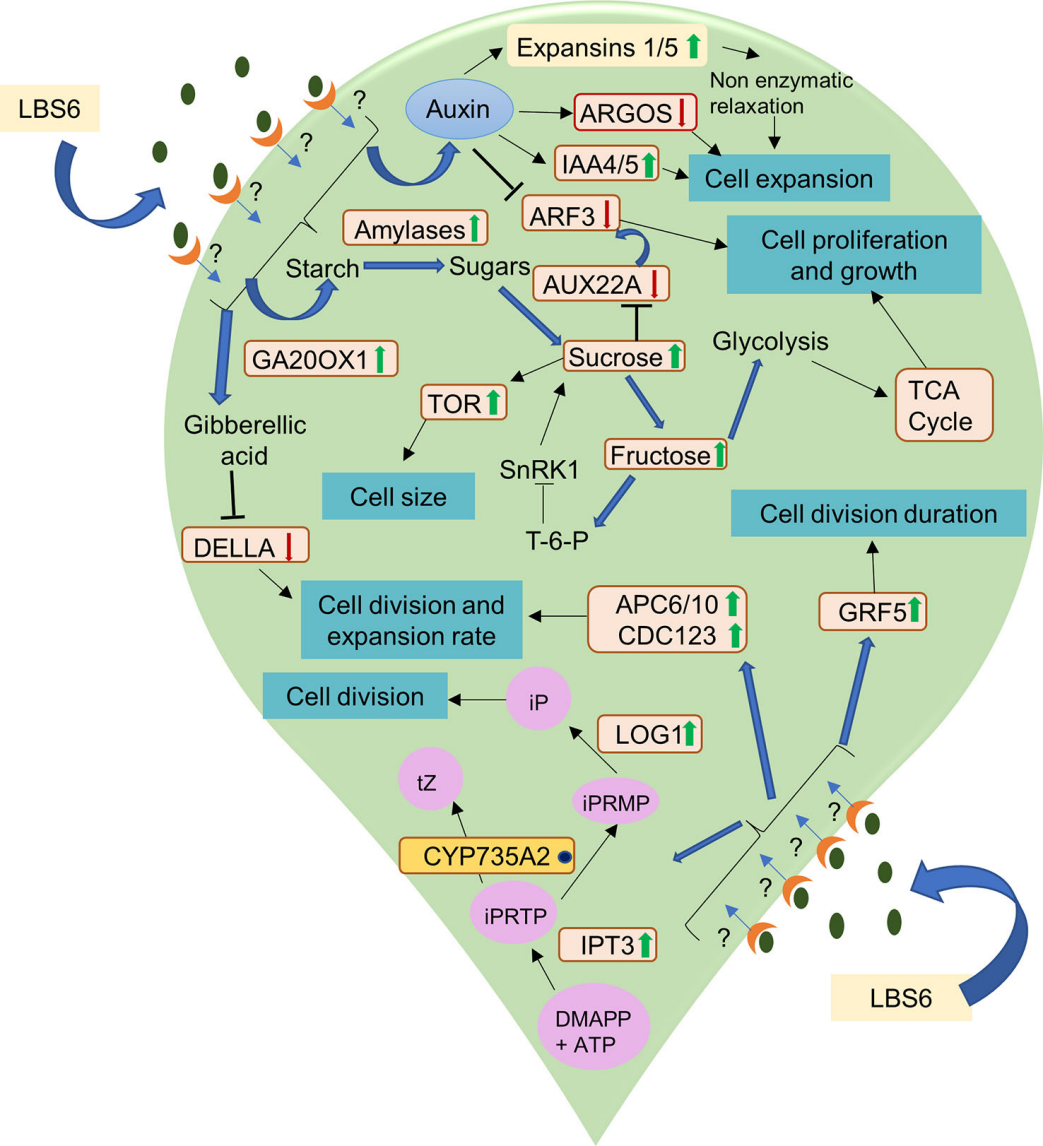
A schematic representation of the mode of action of LBS6 in stimulating cell division, expansion, proliferation in cucumber cotyledons. The green arrow shows the up regulation of the gene expression and enzyme activity, whereas the red arrow shows the downregulation. The blue dot represents no significant change in the expression. Abbreviations listed in the diagram are as follows: *Ga20OX1* (gibberellin 20-oxidase 1), *ARGOS* (auxin regulated gene involved in organ size), *IAA* (indole acetic A inducible gene), TOR (target of rapamycin), *ARF* 3(auxin response factors 3), *Aux22A* (auxin induced protein 22A), *APC6* (anaphase promoting complex 6), *APC10* (anaphase promoting complex 10), CDC123 (cell division cycle protein 123 homolog), *LOG1* (Lonely guy 1), *GRF5* (growth regulating factor 5), DMAPP (dimethylallyl diphosphate), iPRTP (N6 -(2-isopentenyl Δ) adenine riboside 5′-triphosphate), iPRMP (N^6^ -(2-isopentenyl Δ) adenine riboside 5′-monophosphate), iP (N6 -(D2 -isopentenyl)adenine), tZ (*trans*-zeatin).

Cytokinin is synthesized in plants by conversion of 5-phosphate adenosine (AMP, ATP, and ADP) to N6 -(2-isopentenyl Δ) adenine riboside 5′-triphosphate (iPRTP) by adenosine phosphate isopentenyl-transferase (IPT3) (Li et al., 2021). The iPRTP is further phosphorylated to form N^6^ -(2-isopentenyl Δ) adenine riboside 5′-monophosphate (iPRMP), which is then hydroxylated to form tZ-type cytokinin by CYP735A1/CYP735A2 of the cytochrome P450 monooxygenase (P450) family (Li et al., 2021). LBS6 modulated the cytokinin production in the cotyledons, which was evident based on expression of key genes involved in cytokinin metabolism. *IPT3*, a cytokinin biosynthetic gene, was induced earlier in the LBS6-treated cotyledons (24 h) than in the control (48 h), suggesting higher cytokinin during the early cell division phase 24 h after the treatment was applied. *CYP735A2* was found to be down-regulated in both control and LBS6-treated cotyledons. No significant change was observed between control and LBS6 treated cotyledons. Lonely Guy 1 (*LOG1*), an additional cytokinin biosynthetic gene converts iPRMP to iP (N6 -(D2 -isopentenyl) adenine (iP)) (Kurakawa et al., 2007), was found to be significantly up-regulated in LBS6 treatments, suggesting that the red seaweed extract induced the conversion of iPRMP to iP *via LOG1*, rather than iPRMP to tZ-type cytokinin *via CYP735A2. LOG1* plays the role of fine coordination between bioactive cytokinin and cell division (Kurakawa et al., 2007). Cytokinin oxidases/dehydrogenases (CKX) are involved in the non-reversible degradation of bioactive cytokinin (iP, tZ) (Schmülling et al., 2003). Cytokinin dehydrogenases and *ARR5* were also induced in the LBS6-treated cotyledons, after 72 h, showing that LBS6 negatively regulated cytokinin metabolism. These results showed that application of the LBS6 extract mediated cotyledon expansion by regulating the cytokinin biosynthetic genes. Thus, to conclude, the LBS6-treated cotyledons balance the fine tuning between cell development and proliferation, and phytohormone and sugar metabolism in order to induce expansion in the cotyledon (**Figure 12**).

### 4.2 Plant growth assay

The cucumber cotyledon expansion bioassay demonstrates the beneficial effects of LBS6 applications, but ideally it should be translated to a whole plant assay. Foliar application of LBS6 was tested on cucumber plants, grown under hydroponic conditions. LBS6-spray treated plant produced significantly higher leaf area, as compared to control. This phenotype can be correlated with the expansion of the cotyledon. Additionally, sprayed plants showed significant impacts on root architecture. The root length, surface area and volume were significantly higher in those treated plants. Fresh and dry biomass of shoots and roots were also significantly higher than their control counterparts. Similarly, Trivedi et al. (2017) also showed that their particular *Kappaphycus*-derived phyco-biostimulant prepared (differently than LBS6) improved the leaf area of maize. Potter and Jones (1977) showed that relative leaf area expansion was directly correlated with rates of photosynthesis. Various components of the LBS6 applications therefore played important roles in regulating leaf size, and by extension, enhanced photosynthetic area of the primary leaves which could be directly associated with higher photosynthetic capacity (Hu et al., 2020).

In this study, the effect of the LBS6 extract on photosynthetic parameters was assayed using chlorophyll fluorescence (Maxwell and Johnson, 2000). Chlorophylls are involved in the trapping of light energy, as an excitation energy for fixing atmospheric CO_2_ (Kandel, 2020). Measurement of SPAD values represents chlorophyll content (Kandel, 2020). The spray application of LBS6 showed significant increases in SPAD values, suggesting a higher chlorophyll content and hence more absorption capacity of light energy. An increase in PSII activity was observed in LBS6-sprayed plants in terms of the relative activity of PSII (Fv’/Fm’), the fraction of PSII open center (qL) and the quantum yield of PSII electron transport (Phi2). However, these incremental values were not statistically significant. These data suggested that little change was observed in excitement of the PSII biochemistry in either treatments or controls. Whilst more active, open, and oxidized PSI were present in LBS6- sprayed plants, as compared to control. The loss of dissipation of energy due to non-photochemical quenching (PhiNPQ) was found to be significantly reduced in LBS6-treated plants, thereby suggesting that the commercial extract was indeed helping treated plants to efficiently use photochemical energy for CO_2_ fixation.

The photosynthetically active radiation (PAR) absorbed by reaction centers of PSII generates the electron flow through the different electron receptor in the thylakoid membrane for generation of ATP and NADPH, which are used during Calvin cycle for CO_2_ fixation (Huang et al., 2015; Yamori et al., 2015). This is an important parameter that can be correlated to plant growth and stress tolerance (Ibrahimova et al., 2021). Higher ETR_PSII_ in the LBS6-sprayed plants helps PSII to efficiently extract electron and protons from the water, which eventually leads to higher generation of ATP and NADPH for increased CO_2_ fixation during the Calvin cycle. The dark interval relaxation kinetics (DIRK) of the electrochromic band shift measured by the MultispeQ was used to determine proton motive force (ECSt), proton conductance of chloroplast ATP synthase (gH^+^), and estimated proton flux through thylakoid lumen (vH^+^) which represents rate of ATP synthesis (Avenson et al., 2004; Kanazawa et al., 2017; Ibrahimova et al., 2021). LBS6 treatments showed a reduction in ECSt which was associated with increase of gH^+^. These results clearly explained the role of LBS6 applications in improving photosynthesis by increasing the ETR_PSII,_ which results in decreased non-photochemical quenching, by increasing the pH of the thylakoid lumen. Increased ETR_PSII_ in LBS6 treatments resulted in higher assimilation, which in turn, increased demand for ATP. To meet the demands of higher ATP, LBS6-sprayed plants produced a higher proton conductance of chloroplast ATP synthase (gH^+^), and estimated proton flux through the thylakoid lumen (vH^+^), which was translated to higher rate of ATP synthesis (Kanazawa et al., 2017). These results provide clear evidence that LBS6, derived from *K. alvarezii*, improved the plant growth by improving photosynthetic efficiency.

## 5 Conclusion

The bioactive components present in this proprietary extraction of the commercially valuable *Kappaphycus alvarezii* – a phyco-biostimulant was shown to improve the growth of treated cotyledons of cucumber by regulating cell proliferation and expansion. This study, using the cucumber cotyledon expansion as a bioassay, provides a holistic molecular insight on the mode of action of foliar spray of LBS6 through which it improves growth, expansion and proliferation of cells in plants. The treatment of LBS6 modulated the expression of genes involved in cell division, expansion, proliferation and growth of cucumber cotyledon. The foliar spray of LBS6 on cucumber plants grown under hydroponic conditions showed higher shoot and root biomass. Further, the foliar application of the LBS6 modulated both electron and proton transport-related pathways which helped the treated plants to efficiently utilize the photosynthetic radiation for optimal plant growth. This study provides concurrent evidence that the use of a *Kappaphycus*-based biostimulant regulated developmental, cellular, physiological, and molecular pathways to promote plant growth. The results presented in this study provide significant additions to knowledge on the varied modes of actions of red seaweed derived unique biostimulant (LBS6)-mediated plant growth. This study provides the basis for usage of LBS6 as a sustainable strategy for improving the productivity of important plant crops. However, further studies are required to decipher role of bioactives of LBS6 in stress tolerance, nutrient- and water-use-efficiencies.

## Data availability statement

The raw data supporting the conclusions of this article will be made available by the authors, without undue reservation.

## Author contributions

PS, SKu, SN and SS contributed to conception and design of study. PS and NN designed and performed the experiments. DB contributed to estimation of fructose and sucrose. PS and NN performed data processing, analysis, and interpretation. SKu and SKh develop formulation and created time lapse video, PS wrote the first draft of manuscript. NN wrote section of manuscript. PS, AC, SKu and SS contributed to the final versions of manuscript. All authors contributed to the article and approved the submitted version.
