# Supplementary material for: Understanding the mode of action of AgroGain®, a biostimulant derived from the red seaweed Kappaphycus alvarezii in the stimulation of cotyledon expansion and growth of Cucumis sativa (cucumber)
Source: Front Plant Sci. 2023 Apr 6;14:1136563. doi: 10.3389/fpls.2023.1136563 (PMC10118050; doi:10.3389/fpls.2023.1136563)
Supplement: Supplementary file 2 [file DataSheet_1.pdf]

*Supplementary Material*

# Understanding the mode of action of AgroGain®, a biostimulant derived from the red seaweed *Kappaphycus alvarezii* in the stimulation of cotyledon expansion and growth of *Cucumis sativa* (cucumber)

**Pushp Sheel Shukla[1*], Nagarajan Nivetha[1], Sri Sailaja Nori[1*], Debayan Bose[1], Sawan Kumar[1], Sachin Khandelwal[1], Alan Critchley[2], Shrikumar Surynaryanan[1]**

*** Correspondence:**
Pushp Sheel Shukla
pushp.shukla@sea6energy.com
Sri Sailaja Nori
sailaja@sea6energy.com

**Supplementary Table S1: Chemical composition of LBS06**

| S.No | Parameters | Concentration |
|---|---|---|
| 1 | Dry matter | 23 % |
| 2 | Total Nitrogen (N) | < 0.100 % |
| 3 | Phosphorus ($P_2O_5$), mineral acid soluble | 0.04 % |
| 4 | Potassium ($K_2O$), mineral acid soluble | 7.25 % |
| 5 | Potassium ($K_2O$), water-soluble | 7.07 % |
| 6 | Magnesium, total (calculated as MgO) | 0.29 % |
| 7 | Calcium, total (calculated as CaO) | 0.43 % |
| 8 | Total Sulphur (S) | 0.81 % |
| 9 | Sodium (Na) | 1.13 % |
| 10 | Ash | 15.48 % |
| 11 | Organic matter | 7.49 % |
| 12 | Alkaline active components (calculated as CaO) | < 0.0100 % |
| 13 | Cobalt (Co) | 0.83 mg/kg |
| 14 | Copper (Cu) | < 2.00 mg/kg |
| 15 | Selenium (Se) | < 0.200 mg/kg |
| 16 | Zinc (Zn) | 6.35 mg/kg |
| 17 | pH value | 4 |
| 18 | Boron (B) | 22.1 mg/kg |

## Supplementary Table S2: List of the primes used in the study

| Gene name (Accession number) | Primer sequence | |
|---|---|---|
| **CsAPC6 F (Csa_4G506830)** | TTGCTGTCGGCTGCTATTAC | **Cell Division rate** |
| **CsAPC6 R (Csa_4G506830)** | CAAGCAGGTGCAAATGTTCC | |
| **CsCDC123 F (Csa_1G571790)** | AATCCATGGGGTGCGTTTAC | |
| **CsCDC123 R (Csa_1G571790)** | TCTTCAACCCTGGTCTCACTG | |
| **CsAPC10 F (Csa_3G839820)** | GCTGCAAATTGCCGTTCTATC | |
| **CsAPC10 R (Csa_3G839820)** | CGGTCGAGGTCCATAGATTTG | |
| **CsARF3 F (Csa_6G518210)** | GCATGTGTCCCACTTCATTGG | **Cell Number** |
| **CsARF3 R (Csa_6G518210)** | TGTAAACGCTGCTGATGCTG | |
| **CsDELLA F (Csa_5G569350)** | TTGAATCGGTGGTGGTGAAC | **Cell Expansion rate** |
| **CsDELLA R (Csa_5G569350)** | TGATTCGCTTCTTGCTCCAC | |
| **CsARGOS like F (Csa_2G123610)** | TTTGATGCTGCTGCTGCTTC | **Cell Size** |
| **CsARGOS like R (Csa_2G123610)** | AGAAGGCATAAACGCTAACACC | |
| **CsTOR F (Csa_3G811590)** | TGTGAACCCCATCGATACCTTG | |
| **CsTOR R (Csa_3G811590)** | CGTGAATCCATCAACGTTCCAG | |
| **Cs Expansin A5 F (Csa_5G636630)** | TTCCTGGCACCATTGTTGTC | |
| **Cs Expansin A5 R (Csa_5G636630)** | AATCCCAGCGCGATATTGAG | |
| **Cs Expansin A1 F (Csa_5G605760)** | AAGAGGAGGCATAAGGTTCACC | |
| **Cs Expansin A1 R (Csa_5G605760)** | CCCAGTTCCTTGACATTGCTTG | |
| **CsGa20O1D F (Csa_5G172270)** | GGTGAACAGCAAGAGAACAAGG | |
| **CsGa20O1D R (Csa_5G172270)** | GTTCTCATTGTCCACCAACTCC | |
| **CsGRF5F (Csa_3G651860)** | TAAACAGGCAAGGCAACAGC | |
| **CsGRF5R (Csa_3G651860)** | GCTTCTGGGTTTCTGCATCTTC | |
| **CsUBQ5 F** | GACCAGCAGCGTTTGATTTTC | **Reference gene** |
| **CsUBQ5 R** | CTTGGGCTTGGTGTAGGTCTTC | |

| Gene name (Accession number) | Primer sequence | |
|---|---|---|
| **CsAPC6 F (Csa_4G506830)** | TTGCTGTCGGCTGCTATTAC | Cell Division rate |
| **CsAPC6 R (Csa_4G506830)** | CAAGCAGGTGCAAATGTTCC | |
| **CsCDC123 F (Csa_1G571790)** | AATCCATGGGGTGCGTTTAC | |
| **CsCDC123 R (Csa_1G571790)** | TCTTCAACCCTGGTCTCACTG | |
| **CsAPC10 F (Csa_3G839820)** | GCTGCAAATTGCCGTTCTATC | |
| **CsAPC10 R (Csa_3G839820)** | CGGTCGAGGTCCATAGATTTG | |
| **CsARF3 F (Csa_6G518210)** | GCATGTGTCCCACTTCATTGG | Cell Number |
| **CsARF3 R (Csa_6G518210)** | TGTAAACGCTGCTGATGCTG | |
| **CsDELLA F (Csa_5G569350)** | TTGAATCGGTGGTGGTGAAC | Cell Expansion rate |
| **CsDELLA R (Csa_5G569350)** | TGATTCGCTTCTTGCTCCAC | |
| **CsARGOS like F (Csa_2G123610)** | TTTGATGCTGCTGCTGCTTC | Cell Size |
| **CsARGOS like R (Csa_2G123610)** | AGAAGGCATAAACGCTAACACC | |
| **CsTOR F (Csa_3G811590)** | TGTGAACCCCATCGATACCTTG | |
| **CsTOR R (Csa_3G811590)** | CGTGAATCCATCAACGTTCCAG | |
| **Cs Expansin A5 F (Csa_5G636630)** | TTCCTGGCACCATTGTTGTC | |
| **Cs Expansin A5 R (Csa_5G636630)** | AATCCCAGCGCGATATTGAG | |
| **Cs Expansin A1 F (Csa_5G605760)** | AAGAGGAGGCATAAGGTTCACC | |
| **Cs Expansin A1 R (Csa_5G605760)** | CCCAGTTCCTTGACATTGCTTG | |
| **CsGa20O1D F (Csa_5G172270)** | GGTGAACAGCAAGAGAACAAGG | |
| **CsGa20O1D R (Csa_5G172270)** | GTTCTCATTGTCCACCAACTCC | |
| **CsGRF5F (Csa_3G651860)** | TAAACAGGCAAGGCAACAGC | |
| **CsGRF5R (Csa_3G651860)** | GCTTCTGGGTTTCTGCATCTTC | |
| **CsUBQ5 F** | GACCAGCAGCGTTTGATTTTC | Reference gene |
| **CsUBQ5 R** | CTTGGGCTTGGTGTAGGTCTTC | |
